# Senses along Which the Entropy *S_q_* Is Unique

**DOI:** 10.3390/e25050743

**Published:** 2023-05-01

**Authors:** Constantino Tsallis

**Affiliations:** 1 Centro Brasileiro de Pesquisas Físicas and National Institute of Science and Technology of Complex Systems, Rua Xavier Sigaud 150, Rio de Janeiro 22290-180, RJ, Brazil; tsallis@cbpf.br; 2 Santa Fe Institute, 1399 Hyde Park Road, Santa Fe, NM 87501, USA; 3 Complexity Science Hub Vienna, Josefstädter Strasse 39, 1080 Vienna, Austria

**Keywords:** Boltzmann–Gibbs statistical mechanics, nonadditive entropies, nonextensive statistical mechanics, entropic uniqueness theorems

## Abstract

The Boltzmann–Gibbs–von Neumann–Shannon *additive* entropy $S_{BG}=-k\sum_{i}p_{i}\ln p_{i}$ as well as its continuous and quantum counterparts, constitute the grounding concept on which the BG statistical mechanics is constructed. This magnificent theory has produced, and will most probably keep producing in the future, successes in vast classes of classical and quantum systems. However, recent decades have seen a proliferation of natural, artificial and social complex systems which defy its bases and make it inapplicable. This paradigmatic theory has been generalized in 1988 into the *nonextensive statistical mechanics*—as currently referred to—grounded on the *nonadditive* entropy $S_{q}=k\frac{1-\sum_{i}p_{i}^{q}}{q-1}$ as well as its corresponding continuous and quantum counterparts. In the literature, there exist nowadays over fifty mathematically well defined entropic functionals. $S_{q}$ plays a special role among them. Indeed, it constitutes the pillar of a great variety of theoretical, experimental, observational and computational validations in the area of complexity—*plectics*, as Murray Gell-Mann used to call it. Then, a question emerges naturally, namely *In what senses is entropy $S_{q}$ unique?* The present effort is dedicated to a—surely non exhaustive—mathematical answer to this basic question.

## 1. Introduction

Boltzmann–Gibbs (BG) statistical mechanics can arguably be considered as one of the pillars of contemporary theoretical physics, together with Maxwell electromagnetism, Newtonian and quantum mechanics, and Einstein’s special and general relativity. Consistently, the concepts of entropy and energy provide the basis of classical thermodynamics [1]. The BG theory is grounded on the well known BG entropy [2,3,4,5,6], which is additive. The 1988 proposal [7] of nonadditive entropies as a basis to generalize the traditional BG theory led to what is currently referred to as *nonextensive statistical mechanics*. Let us briefly review here the basic issues.

BG statistical mechanics is constructed upon the following Boltzmann–Gibbs–von Neumann–Shannon entropic functional:

(1) $$S_{BG}=-k\sum_{i=1}^{W}p_{i}\ln p_{i}\;\;\left(\sum_{i=1}^{W}p_{i}=1\right)\;,$$

where *k* is a conventional positive constant chosen once for ever (typically $k=k_{B}$ in physics, and k=1 in computational sciences). Its maximal value occurs for equal probabilities, i.e., $p_{i}=1/W\;,\forall i$, and is given by

(2) $$S_{BG}=k\ln W\;,$$

carved on the tombstone of Ludwig Boltzmann in Vienna. This relation constitutes a genius connection between the macroscopic and the microscopic descriptions of real systems. The entropy (1) is *additive* [8]. Indeed, if *A* and *B* are two *probabilistically independent* systems [i.e., $p_{ij}^{A+B}=p_{i}^{A}p_{j}^{B}\;,\forall \left(i,j\right)$], we straightforwardly verify that

(3) $$S_{BG}\left(A+B\right)=S_{BG}\left(A\right)+S_{BG}\left(B\right)\;.$$

Further, for a system in thermodynamical equilibrium with a thermostat at temperature *T*, the distribution which optimizes $S_{BG}$ is given by the celebrated BG weight

(4) $$p_{i}=\frac{e^{-\beta E_{i}}}{\sum_{j=1}^{W}e^{-\beta E_{j}}}\;,$$

where β=1/kT and $\left\{E_{i}\right\}$ are the possible energies of the system.

A generalization of this theory was proposed in 1988 [7] on the basis of the entropic functional

(5) $$S_{q}=k\frac{1-\sum_{i=1}^{W}p_{i}^{q}}{q-1}\;\;\left(q\in R;S_{1}=S_{BG}\right)\;.$$

This functional can also be written as

(6) $$S_{q}=k\sum_{i=1}^{W}p_{i}\ln_{q}\frac{1}{p_{i}}=-k\sum_{i=1}^{W}p_{i}^{q}\ln_{q}p_{i}=-k\sum_{i=1}^{W}p_{i}\ln_{2-q}p_{i}\;,$$

where the *q*-logarithmic function is defined as

(7) $$\ln_{q}z\equiv \frac{z^{1-q}-1}{1-q}\;\;\;\left(\ln_{1}z=\ln z\right)\;.$$

The extremal value of $S_{q}$ is given by the generalization of Equation (2), namely

(8) $$S_{BG}=k\frac{W^{1-q}-1}{1-q}\equiv k\ln_{q}W\;.$$

This value corresponds to a maximum for q>0, to a minimum for q<0, and to the constant k(W−1) for q=0.

Equation (3) is generalized as follows:

(9) $$\frac{S_{q}\left(A+B\right)}{k}=\frac{S_{q}\left(A\right)}{k}+\frac{S_{q}\left(B\right)}{k}+\left(1-q\right)\frac{S_{q}\left(A\right)}{k}\frac{S_{q}\left(B\right)}{k}\;,$$

hence

(10) $$S_{q}\left(A+B\right)=S_{q}\left(A\right)+S_{q}\left(B\right)+\frac{1-q}{k}S_{q}\left(A\right)S_{q}\left(B\right)\;,$$

which recovers Equation (3) in the (q−1)/k→0 limit.

Equation (4) is generalized into

(11) $$p_{i}=\frac{e_{q}^{-\beta_{q}\left(E_{i}-\mu_{q}\right)}}{\sum_{j=1}^{W}e_{q}^{-\beta_{q}\left(E_{j}-\mu_{q}\right)}}\;,$$

where $\mu_{q}$ plays the role of a chemical potential, and $e_{q}\left(x\right)$ is the inverse function of $\ln_{q}x$, i.e.,

(12) $$e_{q}^{x}\equiv {\left[1+\left(1-q\right)x\right]}_{+}^{\frac{1}{1-q}}\;,$$

${\left[\ldots \right]}_{+}$ being equal to […] if […]>0 and zero otherwise; it satisfies $e_{q}^{x}\;e_{2-q}^{-x}=1$.

Details related to this *q*-generalized statistical mechanics, currently referred to as *nonextensive statistical mechanics*, are available at [9,10,11,12], and strong experimental validations are presented in [13,14,15], to cite but a few; full bibliography is available at [16]. In connection with Equation (11), see also [17], where it is shown that, through a Moebius group, one can find a Casimir invariant, which allows to define an observable *inverse temperature* $\beta_{q}$. Let us also mention at this point that exponential and logarithmic deformed functions that extend the *q*-exponential and the *q*-logarithmic ones [18] are already available in the literature [19,20,21].

## 2. On Uniqueness

**Figure 1:**
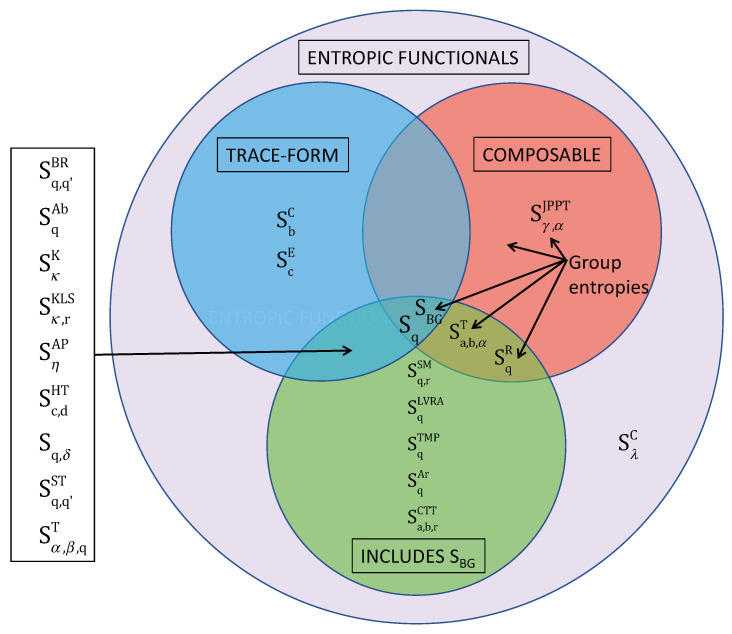
It has been proven [23] that $S_{q}$ is the unique entropic form which simultaneously is trace-form, composable, and recovers $S_{BG}$ as a particular instance. $S_{q}$ (hence $S_{BG}$), the Renyi entropy $S_{q}^{R}$ [24], the Tempesta (a,b,α)-entropy $S_{a,b,\alpha }^{T}$ (Equation (9.1) in [25]), the Jensen–Pazuki–Pruessner–Tempesta entropy $S_{\gamma ,\alpha }^{JPPT}$ [26] and many others belong to the class of *group entropies* and are therefore composable. To facilitate the identification, we are here using the following notations: Sharma–Mittal entropy $S_{q,r}^{SM}$ [27], Landsberg-Vedral-Rajagopal-Abe entropy $S_{q}^{LVRA}$ [28,29,30], Tsallis–Mendes–Plastino entropy $S_{q}^{TMP}$, Arimoto entropy $S_{q}^{Ar}$ [31], Curado–Tempesta–Tsallis entropy $S_{a,b,r}^{CTT}$ [32], Borges–Roditi entropy $S_{q,q^{\prime }}^{BR}$ [33], Abe entropy $S_{q}^{Ab}$ [34], Kaniadakis entropy $S_{\kappa }^{K}$ [35], Kaniadakis–Lissia–Scarfone entropy $S_{\kappa ,r}^{KLS}$ [36], Anteneodo–Plastino entropy $S_{\eta }^{AP}$ [37], Hanel–Thurner entropy $S_{c,d}^{HT}$ [38,39], $S_{q,\delta }$ [40], Schwammle–Tsallis entropy $S_{q,q^{\prime }}^{ST}$ [41], the Tempesta (α,β,q)-entropy $S_{\alpha ,\beta ,q}^{T}$ [42], the Curado *b*-entropy $S_{b}^{C}$ [43,44], the Curado λ-entropy $S_{\lambda }^{C}$ [45] (see [12]), and the exponential *c*-entropy $S_{c}^{E}$ (see [10,46]). The entropic form $S_{\lambda }^{C}$ is one among the rare cases which do not include $S_{BG}$ and is neither trace-form nor composable. From [1,12].

Since the introduction of $S_{BG}$ in the XIX-th century, very many (nearly fifty) entropic functionals have emerged in the literature for a variety of informational, cybernetic, physical, mathematical reasons: see, for instance, Figure 1. For a meticulous listing of existing entropic functionals discussed from a chronological and logical perspective, see [22]; for further historical remarks, see [12] in Section 3.2.1.

$S_{q}$ and, naturally, its particular case $S_{BG}$ reveal some sort of special role within this ever increasingly long list, in the sense that they repeatedly appear, either directly or indirectly through their consequences, in a plethora of natural, artificial and social systems, especially those within which nonlinear dynamics is involved. The present effort is dedicated to review in what senses $S_{q}$ is nowadays known to be unique.

Before focusing on this task, let us mention that, among the many existing extensions of $S_{BG}$, only one is presently known to be additive, all the others being nonadditive. This exception is the Renyi entropic functional [24], defined as follows:

(13) $$S_{q}^{R}=k\frac{\ln\sum_{i=1}^{W}p_{i}^{q}}{1-q}\;\;\left(q\in R;S_{1}^{R}=S_{BG}\right)\;.$$

The nonadditive $S_{q}$ and the additive $S_{q}^{R}$ are related through the following monotonically increasing function [7]:

(14) $$S_{q}^{R}=k\frac{\ln[1+\left(1-q\right)S_{q}/k]}{1-q}\;\;\;\left(\forall q\right).$$

It immediately follows that the extremization of $S_{q}^{R}$ and $S_{q}$ for the same set of constraints yields the same optimizing distribution. For instance, if a value for q<1 exists for a specific class of systems, such that $S_{q}\left(N\right)$ is extensive, i.e., $S_{q}\left(N\right)\propto N$ (N→∞), then $S_{q}^{R}\propto \ln N$. Such a nonlinear asymptotic behavior makes $S_{q}^{R}$ to violate thermodynamical entropic extensivity, which violates in turn the mathematical Legendre structure upon which classical thermodynamics is based. For different purposes, however, the Renyi entropic functional exhibits some interesting mathematical properties (see [47] and references therein). Let us finally mention that the functional relationship (14) plays a central role in recently *q*-generalized mathematical objects [48].

### 2.1. Santos 1997 Theorem

Shannon formulated in 1948 a definitively relevant theorem [49,50], which we summarize here.

Let us assume that an entropic form $S(\left\{p_{i}\right\})$ satisfies the following properties:

(15) $$\begin{array}{ccc} & \left(i\right) & S\left(\left\{p_{i}\right\}\right)\;is\;a\;\;continuous\;function\;of\;\left\{p_{i}\right\}; \end{array}$$

(16) $$\begin{array}{ccc} & \left(ii\right) & S(p_{i}=1/W,\forall i)\;monotonically\;increases\;\;with\;\;the\;\;total\\ & & number\;of\;possibilities\;W; \end{array}$$

(17) $$\begin{array}{ccc} & \left(iii\right) & S\left(A+B\right)=S\left(A\right)+S\left(B\right)\;\;\;\;if\;\;\;p_{ij}^{A+B}=p_{i}^{A}p_{j}^{B}\;\forall \left(i,j\right)\;,\\ & & where\;\;S\left(A+B\right)\equiv S\left(\left\{p_{ij}^{A+B}\right\}\right),\;S\left(A\right)\equiv S\left(\left\{p_{i}^{A}\right\}\right)\;\;\left(p_{i}^{A}\equiv \sum_{j=1}^{W_{B}}p_{ij}^{A+B}\right)\;,\\ & & and\;\;\;\;S\left(B\right)\equiv S\left(\left\{p_{j}^{B}\right\}\right)\;\;\left(p_{j}^{B}\equiv \sum_{i=1}^{W_{A}}p_{ij}^{A+B}\right)\;; \end{array}$$

(18) $$\begin{array}{ccc} & \left(iv\right) & S\left(\left\{p_{i}\right\}\right)=S\left(p_{L},p_{M}\right)+p_{L}S\left(\left\{p_{i}/p_{L}\right\}\right)+p_{M}S\left(\left\{p_{i}/p_{M}\right\}\right)\;\\ & & with\;\;p_{L}\equiv \sum_{L\;terms}p_{i}\;\;,\;\;p_{M}\equiv \sum_{M\;terms}p_{i}\;\;,\\ & & L+M=W\;,\;and\;\;p_{L}+p_{M}=1\;. \end{array}$$

Then and only then [49,50] $S(\left\{p_{i}\right\})$ is given by Equation (1).

It is therefore very clear in what sense the functional (1) is unique, namely that the axiomatic set (i)–(iv) is mathematically equivalent to the functional (1). This neatly differs from the definitively wrong, and yet not rare, statement that form (1) is the unique *physically admissible* entropic functional. Axiom (iv) is sometimes referred to as the *grouping property*. Let us also mention that some authors prefer the notation S(A×B) instead of S(A+B) in order to emphasize the fact that the phase-space of the total system is the tensor product of the space-phases of the subsystems *A* and *B*.

In 1997, Santos theorem [51] generalized that of Shannon as follows:

Let us assume that an entropic form $S(\left\{p_{i}\right\})$ satisfies the following properties:

(19) $$\begin{array}{ccc} & \left(i\right) & S\left(\left\{p_{i}\right\}\right)\;is\;a\;\;continuous\;function\;of\;\left\{p_{i}\right\}; \end{array}$$

(20) $$\begin{array}{ccc} & \left(ii\right) & S(p_{i}=1/W,\forall i)\;monotonically\;increases\;\;with\;\;the\;\;total\\ & & number\;of\;possibilities\;\;W; \end{array}$$

(21) $$\begin{array}{ccc} & \left(iii\right) & \frac{S(A+B)}{k}=\frac{S(A)}{k}+\frac{S(B)}{k}+\left(1-q\right)\frac{S(A)}{k}\frac{S(B)}{k}\;\;\;\;\\ & & if\;\;p_{ij}^{A+B}=p_{i}^{A}p_{j}^{B}\;\forall \left(i,j\right),\;\;with\;\;k>0; \end{array}$$

(22) $$\begin{array}{ccc} & \left(iv\right) & S\left(\left\{p_{i}\right\}\right)=S\left(p_{L},p_{M}\right)+p_{L}^{q}S\left(\left\{p_{i}/p_{L}\right\}\right)+p_{M}^{q}S\left(\left\{p_{i}/p_{M}\right\}\right)\;\\ & & with\;\;p_{L}\equiv \sum_{L\;terms}p_{i}\;\;,\;\;p_{L}\equiv \sum_{M\;terms}p_{i}\;\;,\\ & & L+M=W,\;\;and\;\;p_{L}+p_{M}=1\;. \end{array}$$

Then and only then [51] $S(\left\{p_{i}\right\})$ is given by Equation (5).

### 2.2. The 1997 Connection to Weak Chaos in the Logistic Map

The first connection between the entropy $S_{q}$ and nonlinear dynamical systems, namely the logistic map, was established in 1997 [52]. This connection was analytically complemented one year later [53]. Since then, a vast literature has been dedicated to this connection, which we summarize in what follows.

The logistic map is a paradigmatic one-dimensional dissipative nonlinear dynamical system. It is defined as follows:

(23) $$x_{t+1}=1-ax_{t}^{2}\;\;\;\left(t=0,1,2,\ldots ;\;a\;\in \left[0,2\right];\;x_{t}\in \left[-1,1\right]\right).$$

For a=2, the system is *strongly chaotic*, the *sensitivity to the initial conditions* is given by $\xi \equiv \lim_{\Delta x_{0}\to 0}\frac{\Delta x_{t}}{\Delta x_{0}}=e^{\lambda_{1}\;t}$, the Lyapunov exponent $\lambda_{1}$ being equal to ln2=0.69…, and its *entropy production per unit time* is given by the Pesin identity (see details in [54] and references therein)

(24) $$K_{BG}\equiv \lim_{t\to \infty }\frac{S_{BG(t)}}{t}=\lambda_{1}\;,$$

where the subindex 1 will become clear here below.

At the Feigenbaum point $a_{c}=1.40115518909205\ldots$, the system is *weakly chaotic*, the Lyapunov exponent $\lambda_{1}$ vanishes, the *sensitivity to the initial conditions* is given by $\xi =e_{q}^{\lambda_{q}\;t}$, the *q*-generalized Lyapunov coefficient $\lambda_{q}$ being described in [55], and its *q*-generalized entropy production per unit time is given by the Pesin-like identity (see details in [54] and references therein)

(25) $$K_{q}\equiv \lim_{t\to \infty }\frac{S_{q}\left(t\right)}{t}=\lambda_{q}\;,$$

where q=0.24448770134128…

These remarkable results by no means prove, on rigorous mathematical grounds, the uniqueness of $S_{q}$ in what concerns such connections with say generic dissipative nonlinear one-dimensional dynamical systems. For example, the Kaniadakis entropy also implies a *finite* slope $\lim_{t\to \infty }\left[S_{\kappa }^{K}\left(t\right)/t\right]$; this is in fact not surprising since the Kaniadakis entropy is a linear combination of $S_{q}$’s. However, to the best of our knowledge, no other entropic functional but $S_{q}$ has been shown to lead to a basic relation such as (25).

### 2.3. Connection with Jackson Derivative

We follow here along the lines of [12]. One century ago, the mathematician Jackson generalized [56,57] the concept of *derivative* of a generic function f(x). He introduced his differential operator $D_{q}$ as follows:

(26) $$D_{q}f\left(x\right)\equiv \frac{f(qx)-f(x)}{qx-x}\;.$$

We immediately verify that $D_{1}f\left(x\right)=df\left(x\right)/dx$. For q≠1, this operator replaces the usual (infinitesimal) *translation* operation on the abscissa *x* of the function f(x) by a *dilatation* operation.

Abe noticed in 1997 a remarkable property [34] which uniquely yields $S_{q}$. In the same way that we can easily verify that

(27) $$S_{BG}=-\frac{d}{dx}\;\sum_{i=1}^{W}p_{i}^{\;x}{|}_{x=1}\;,$$

we can verify that, ∀q,

(28) $$S_{q}=-D_{q}\;\sum_{i=1}^{W}p_{i}^{\;x}{|}_{x=1}\;.$$

This is an interesting property, where the usual *infinitesimal* translational operation is replaced by a *finite* operation, namely, in this case, by the one which is basic for *scale-invariance*. This fact is in some sense consistent with the definition of the entropy $S_{q}$, which was inspired [7] by multifractal geometry.

### 2.4. Abe 2000 Theorem

In 1953, Khinchin uniqueness theorem [58] further reformulated that of Shannon in a very elegant manner:

Let us assume that an entropic form $S(\left\{p_{i}\right\})$ satisfies the following properties:

(29) $$\begin{array}{ccc} & \left(i\right) & S\left(\left\{p_{i}\right\}\right)\;\;is\;\;a\;\;\;continuous\;\;function\;\;of\;\left\{p_{i}\right\}; \end{array}$$

(30) $$\begin{array}{ccc} & \left(ii\right) & S(p_{i}=1/W,\forall i)\;monotonically\;\;increases\;\;with\;\;the\;\;total\\ & & number\;\;of\;\;possibilities\;\;W; \end{array}$$

(31) $$\begin{array}{ccc} & \left(iii\right) & S\left(p_{1},p_{2},\ldots ,p_{W},0\right)=S\left(p_{1},p_{2},\ldots ,p_{W}\right); \end{array}$$

(32) $$\begin{array}{ccc} & \left(iv\right) & S(A+B)=S(A)+S(B|A)\;,\;\;\;\\ & & where\;\;S\left(A+B\right)\equiv S\left(\left\{p_{ij}^{A+B}\right\}\right),\;S\left(A\right)\equiv S\left(\left\{p_{i}^{A}\right\}\right)\;\;\left(p_{i}^{A}\equiv \sum_{j=1}^{W_{B}}p_{ij}^{A+B}\right)\;,\\ & & and\;\;the\;\;conditional\;\;entropy\;\;S\left(B|A\right)\equiv \sum_{i=1}^{W_{A}}p_{i}^{A}S\left(\left\{p_{ij}^{A+B}/p_{i}^{A}\right\}\right)\;. \end{array}$$

Then and only then [59] $S(\left\{p_{i}\right\})$ is given by Equation (1).

It follows then that the Shannon and the Khinchin sets of axioms are mathematically equivalent.

In 2000, the Abe theorem [60] generalized that of Khinchin as follows:

Let us assume that an entropic form $S(\left\{p_{i}\right\})$ satisfies the following properties:

(33) $$\begin{array}{ccc} & \left(i\right) & S\left(\left\{p_{i}\right\}\right)\;\;is\;\;a\;\;\;continuous\;\;function\;\;of\;\left\{p_{i}\right\}; \end{array}$$

(34) $$\begin{array}{ccc} & \left(ii\right) & S(p_{i}=1/W,\forall i)\;monotonically\;\;increases\;\;with\;\;the\;\;total\\ & & number\;\;of\;\;possibilities\;W; \end{array}$$

(35) $$\begin{array}{ccc} & \left(iii\right) & S\left(p_{1},p_{2},\ldots ,p_{W},0\right)=S\left(p_{1},p_{2},\ldots ,p_{W}\right); \end{array}$$

(36) $$\begin{array}{ccc} & \left(iv\right) & \frac{S(A+B)}{k}=\frac{S(A)}{k}+\frac{S(B|A)}{k}+\left(1-q\right)\frac{S(A)}{k}\frac{S(B|A)}{k}\;\;\;\\ & & where\;\;S\left(A+B\right)\equiv S\left(\left\{p_{ij}^{A+B}\right\}\right),\;S\left(A\right)\equiv S\left(\left\{\sum_{j=1}^{W_{B}}p_{ij}^{A+B}\right\}\right),\;and\;\;\;the\;\\ & & conditional\;\;entropy\;\;S\left(B|A\right)\equiv \frac{\sum_{i=1}^{W_{A}}{\left(p_{i}^{A}\right)}^{q}S\left(\left\{p_{ij}^{A+B}/p_{i}^{A}\right\}\right)}{\sum_{i=1}^{W_{A}}{\left(p_{i}^{A}\right)}^{q}}\;\;\left(k>0\right) \end{array}$$

Then and only then [60] $S(\left\{p_{i}\right\})$ is given by Equation (5).

The possibility of existence of such a theorem through the appropriate generalization of Khinchin’ s fourth axiom had already been considered by Plastino and Plastino [61,62]. Abe established [60] the precise form of this generalized fourth axiom, and proved the theorem.

Notice that, interestingly enough, what enters in the definition of the conditional entropy is the escort distribution, and *not* the original one. Notice also that Equation (35) only holds for q>0. Therefore the expression (5) for q<0 can only be defined for *strictly positive* values of $\left\{p_{i}\right\}$, and it is to be understood as an analytical extension of the q>0 case.

Let us finally emphasize that both Santos axioms and Abe axioms are necessary and sufficient conditions for the emergence of $S_{q}$. Consequently, those two sets of axioms are mathematically equivalent.

The axiomatic justification of diverse entropic functionals has in fact deserved great attention in both recent and not so recent literature. Let us summarize here, along lines close to those presented by Jizba and Korbel [63], the present status of this interesting path of research. Three main consistent lines of analysis exist, namely generalized Shannon–Khinchine axioms of the type of [51,60], the Shore and Johnson axioms [64,65,66], and the Uffink class of entropies [67]. All three lead to the same set of admissible entropies, which includes $S_{q}$ (and also, in some formulations, monotonic functions of $S_{q}$). The particular line related to the Shore–Johnson axioms deserves a special attention because it has been the object of a neat controversy, which is focused on in Section 2.10 hereafter.

### 2.5. Beck-Cohen 2003 Superstatistics

An interesting physical interpretation of nonextensive statistics was preliminary advanced in the early 2000s by Wilk and Wlodarczyk [68] and by Beck [69]. This interpretation was beautifully generalized and formalized, in 2003, in what is currently known nowadays as the Beck–Cohen superstatistics [70]. This phenomenological theory generalizes nonextensive statistics in the sense that its generic state distribution contains the *q*-exponential one as a particular case.

Beck and Cohen [70,71,72,73] start from the standard BG exponential factor but with β being itself a random variable (whence the name “superstatistics”) due to possible spatial and/or temporal fluctuations. They define

(37) $$P\left(E\right)=\int_{0}^{\infty }d\beta^{\prime }f\left(\beta^{\prime }\right)\;e^{-\beta^{\prime }E}\;,$$

where $f(\beta^{\prime })$ is a normalized distribution, such that P(E) also is normalizable under the same conditions as the Boltzmann factor $e^{-\beta^{\prime }E}$ itself is. They also define

(38) $$q_{BC}\equiv \frac{\langle {\left(\beta^{\prime }\right)}^{2}\rangle }{{\langle \beta^{\prime }\rangle }^{2}}=\frac{\int_{0}^{\infty }d\beta^{\prime }\;{\left(\beta^{\prime }\right)}^{2}f\left(\beta^{\prime }\right)}{{\left[\int_{0}^{\infty }d\beta^{\prime }\;\beta^{\prime }f\left(\beta^{\prime }\right)\right]}^{2}}\;,$$

where we have introduced *BC* standing for *Beck-Cohen*. Unless $f(\beta^{\prime })$ is deduced from first principles, this theory is a phenomenological one.

If $f\left(\beta^{\prime }\right)=\delta \left(\beta^{\prime }-\beta \right)$ we recover the BG weight

(39) $$P\left(E\right)=e^{-\beta E}\;,$$

and $q_{BC}=1$.

If $f(\beta^{\prime })$ is the $\chi^{2}$-distribution with *n degrees of freedom* (particular case of the Gamma distribution), i.e.,

(40) $$f\left(\beta^{\prime }\right)=\frac{n}{2\beta \Gamma \left(\frac{n}{2}\right)}{\left(\frac{n\beta^{\prime }}{2\beta }\right)}^{n/2-1}\exp\left\{-\frac{n\beta^{\prime }}{2\beta }\right\}\;\;\;\;\;\left(n=1,2,3,\ldots \right)\;,$$

we obtain

(41) $$P\left(E\right)=e_{q}^{-\beta E}\;,$$

with $q_{BC}=q=\frac{n+2}{n}\geq 1$.

In addition to the so-called *$\chi^{2}$-superstatistics* described above, we have the so-called *inverse $\chi^{2}$-superstatistics*, where it is $1/\beta^{\prime }$, instead of $\beta^{\prime }$, that follows the $\chi^{2}$ distribution. Finally, a third class is sometimes focused on in the literature. It is referred to as the *log-normal superstatistics*, and corresponds to the case where $\beta^{\prime }$ is distributed along a log-normal distribution. These three classes are sometimes referred to as *universality* ones because they are all connected to Gaussians, which, in the Central Limit Theorem sense, are attractors in the space of distributions.

Several other examples of $f(\beta^{\prime })$ are discussed in [70], and it is eventually established an important result, namely that *all narrowly peaked distributions $f(\beta^{\prime })$ yield, as its first nontrivial leading order, q-statistics with $q=q_{BC}$.* As we know, the *q*-exponential distribution emerges naturally from extremizing the entropic functional $S_{q}$. Let us however emphasize that this argument does *not* prove a uniqueness sense for $S_{q}$. It nevertheless points towards some special role being played by this entropic functional. Further issues along this line have been studied in [74,75,76,77,78].

### 2.6. Lattice-Boltzmann Models for Fluids

In the present Subsection we closely follow [12]. The incompressible Navier-Stokes equation has been considered, by Boghosian et al. in 2003 [79], on a discretized *D*-dimensional Bravais lattice of coordination number *b*. It is further assumed that there is a single value for the particle mass, and also for speed. The basic requirement for the lattice-Boltzmann model is to be *Galilean-invariant* (i.e., invariant under change of inertial reference frame), like the Navier–Stokes equation itself. It has been proved [79] that an H-theorem is satisfied for a trace-form entropy (i.e., of the form $S\left(\left\{p_{i}\right\}\right)=\sum_{i}^{W}f\left(p_{i}\right)$) only if it has the form of $S_{q}$ with

(42) $$q=1-\frac{2}{D}\;.$$

Therefore q<1 in all cases (q>0 if D>2, q<0 if D<2, and q=0 for D=2), and approaches unity from below in the D→∞ limit. This interesting result has been generalized by allowing multiple masses and multiple speeds. Galilean invariance once again mandates [80] an entropy of the form of $S_{q}$, with a unique value of *q* determined by a transcendental equation involving the dimension and symmetry properties of the Bravais lattice as well as the multiple values of the masses and of the speeds. Of course, Equation (42) is recovered for the particular case of single mass and single speed. Summarizing, under quite general mathematical hypotheses (including the entropic functional to be of the trace-form), the natural imposition of the Galilean invariance for lattice-Boltzmann models for fluids mandates the use of $S_{q}$.

### 2.7. Topsoe 2005 Factorizability in Game Theory

Topsoe proposed [81,82] an abstract zero-sum game theory between two players, namely “Nature”, aiming at high complexity, and “the physicist”, aiming at low complexity. We describe here a simple illustration of the game ingredients; full mathematical details are available in [81,82]. For simplicity, the set of possibilities (*alphabet*) is here assumed discrete and finite, *W* being the number of possibilities. The probability set associated with “Nature” is $P\equiv \{p_{i}\}$ (with $\sum_{i=1}^{W}p_{i}=1$), and that associated with “the physicist” is $Q\equiv \{q_{i}\}$ (with $\sum_{i=1}^{W}q_{i}=1$). The focus is then put on the triple (Φ,S,D), where the *complexity* Φ, the *entropy S*, and the *divergence D* are respectively given by

(43) $$\Phi \left(P||Q\right)=\sum_{i=1}^{W}\left[q_{i}f\left(\frac{p_{i}}{q_{i}}\right)-f\left(p_{i}\right)\right]\;,$$

(44) $$S\left(P\right)=-\sum_{i=1}^{W}f\left(p_{i}\right)\;,$$

and

(45) $$D=\left(P||Q\right)=\sum_{i=1}^{W}q_{i}f\left(\frac{p_{i}}{q_{i}}\right)\;,$$

where the *generator* f(x) is a real-valued analytic and strictly convex function on [0,1] such that f(0)=f(1)=0 and $f^{\prime }\left(1\right)=1$ (normalization condition).

The Topsoe 2005 theorem [82] states: *A complexity function of the form (43) factorizes if and only if it is related to the Tsallis entropic function.*

### 2.8. Amari-Ohara-Matsuzoe 2012 Conformally Invariant Geometry

A information-geometrical approach [83] leads to an abstract uniqueness property that we briefly summarize here. The generalized logarithm defined in Equation (7) is nowadays placed within a more general frame [19,20,21], referred to as χ-logarithm and defined as follows:

(46) $$\ln_{\chi }z\equiv \int_{1}^{z}\frac{dt}{\chi (t)}\;,$$

where χ(t) is a generic function which satisfies simple properties such as being a concave monotonically increasing one; we define consistently the inverse function $e_{\chi }^{z}\equiv \ln_{\chi }^{-1}\left(z\right)$. We straightforwardly verify that $\chi \left(t\right)=t^{q}$ yields $\ln_{\chi }z=\ln_{q}z$ and $e_{\chi }^{z}=e_{q}^{z}$. For χ(t)=t we refer to the *exponential family* and for generic χ(t) we refer to the *deformed exponential family*; naturally, the deformed exponential family includes the exponential one as a particular instance. Many useful concepts such as generalized entropy, divergence and escort probability distribution are associated with each admissible choice of χ(t). In the space of the probability distributions of a vector random variable $x=(x_{1},\ldots ,x_{n})$, two different different types of geometrical structures can be defined from an information-geometrical perspective, namely the *invariant* and the *flat* ones (see details in [83]). The *q*-exponential family is the unique class in the extended class of positive measures, which simultaneously has the invariant and flat geometries. Furthermore, the *q*-family is the unique class of flat geometry that is connected conformally to the invariant geometry.

### 2.9. Enciso–Tempesta 2017 Theorem

In this Section we follow along the lines of [12].

A dimensionless entropic form $S(\left\{p_{i}\right\})$ (i.e., whenever expressed in appropriate conventional units, e.g., in units of *k*) is said *composable* [84,85] (see also [1,10,86,87]) if the entropy S(A+B)/k corresponding to a system composed of two *probabilistically independent* subsystems *A* and *B* can be expressed in the form

(47) $$\frac{S(A+B)}{k}=F\left(\frac{S(A)}{k},\frac{S(B)}{k};\left\{\eta \right\}\right)\;,$$

where F(x,y;{η}) is a smooth function of (x,y) which depends on a (typically small) set of universal indices {η} defined in such a way that F(x,y;{0})=x+y (*additivity*), and which satisfies F(x,0;{η})=x (*null-composability*), F(x,y;{η})=F(y,x;{η}) (*symmetry*), F(x,F(y,z;{η});{η})=F(F(x,y;{η}),z;{η}) (*associativity*). For thermodynamical systems, this associativity appears to be consistent with the 0th Principle of Thermodynamics.

In other words, the whole concept of composability is constructed upon the requirement that the entropy of (A+B) does *not* depend on the microscopic configurations of *A* and of *B*. Equivalently, we are able to macroscopically calculate the entropy of the composed system without any need of entering into the knowledge of the microscopic states of the subsystems. This property appears to be a natural one for an entropic form if we desire to use it as a basis for a statistical mechanics which would naturally connect to thermodynamics.

The entropy $S_{BG}$ is composable since it satisfies Equation (3). In other words, we have $F_{BG}\left(x,y\right)=x+y$. Being $S_{BG}$ nonparametric, no index exists in $F_{BG}$. Further, the Renyi entropy $S_{q}^{R}$ is composable as it satisfies F(x,y)=x+y for all values of *q*. The entropy $S_{q}$ also is composable since it satisfies (9).

Let us also mention that a linear combination of composable entropies is not necessarily composable. Such is the case of the Kaniadakis entropy $S_{\kappa }^{K}$ [35,88,89]. Indeed, it is not composable for all values of κ in spite of being a linear combination of entropies $S_{q}$.

Let us now focus on another relevant property, namely whether an entropic functional is *trace-form*. By definition, an entropy $S(\left\{p_{i}\right\})$ is said *trace-form* if it can be written as $S\left(\left\{p_{i}\right\}\right)=\sum_{i}^{W}f\left(p_{i}\right)$, where f(z) is a generic analytic function in the interval z∈(0,1). Entropies $S_{q}$, $S_{\kappa }^{K}$ and many others are trace-form, in contrast with $S_{q}^{R}$, which is not.

In 2017, Enciso and Tempesta proved [23] that $S_{q}$ is the unique entropic functional being simultaneously composable and trace-form. See Figure 1.

### 2.10. The Shore–Johnson–Axioms Controversy (2005–2019)

To the best of our knowledge, the analysis of $S_{q}$ and its associated thermostatistics was initiated in 2005 [90,91] in connection with the Shore–Johnson axioms for statistical inference [64,65,66].

In 2013, Pressé et al. [92,93,94] started to lengthily insist that the Shore-Johnson axioms exclude entropies such as $S_{q}$. Their arguments were boldly rebutted in [95] (The actual title *Conceptual inadequacy of the Shore and Johnson axioms for wide classes of complex systems* of [95] constitutes a sort of ambiguous shortcut. It should have rather been *Conceptual inadequacy of the Presse et al. interpretation of the Shore and Johnson axioms for wide classes of complex systems*.) where, among other points, it was explicitly written that generic probabilities $\{u_{i}$} and $\left\{v_{j}\right\}$ satisfy, for q≠1,

(48) $$\begin{array}{ccc} \frac{S_{q}\left(\left\{u_{i}{⊗}_{2-q}v_{j}\right\}\right)}{k} & = & -\sum_{ij}\left(u_{i}{⊗}_{2-q}v_{j}\right)\ln_{2-q}\left(u_{i}{⊗}_{2-q}v_{j}\right)=-\sum_{ij}\left(u_{i}{⊗}_{2-q}v_{j}\right)\left(\ln_{2-q}u_{i}+\ln_{2-q}v_{j}\right)\\ & \neq & -\sum_{ij}u_{i}v_{j}\left(\ln_{2-q}u_{i}+\ln_{2-q}v_{j}\right)=-\sum_{i=1}^{W}u_{i}\ln_{2-q}u_{i}-\sum_{j=1}^{W}v_{j}\ln_{2-q}v_{j}\\ & = & \frac{S_{q}\left(\left\{u_{i}\right\}\right)}{k}+\frac{S_{q}\left(\left\{v_{j}\right\}\right)}{k}\;. \end{array}$$

Along their arguments, Presse et al. [92,93,94] definitively violate the imperative inequality present in the middle line of this mathematical chain. They ignore it not only in [92,93,94] but also in their reply [96] to [95]. In fact, the chain (48) is an interesting and nontrivial consequence of this class of correlations (strangely enough, referred in [92] to as “spurious correlations”) between probabilistic events. The fallacies contained in [92,93,94,96] have been meticulously discussed and rebutted in [63,97]. This fact seemingly closes that longstanding controversy, and we are allowed to believe that it is now irreversibly established that $S_{q}$ is admissible within the Shore-Johnson axioms. However, it remains nowadays somewhat unclear whether entropic functionals differing from $S_{q}$ (for instance, $S_{q,\delta }$ or $S_{\kappa }^{K}$) are, as well, admissible within those important axioms. Therefore, with respect to the uniqueness issue, the problem presently appears to be open. For example, it is unknown whether non-trace-form and/or non-composable entropies can satisfy those axioms (or other possible statistical consistency axioms) as well.

### 2.11. Plastino-Tsallis-Wedemann-Haubold 2022

One more sense along which $S_{q}$ is unique has been advanced recently [98].

The BG entropy is defined as the mean value of $\ln\frac{1}{p_{i}}$. Moreover, under the usual linear constraints for the normalization and the energy mean value, it is optimized by the BG exponential factor, which precisely is the inverse function of the logarithmic function. Similarly, the $S_{q}$ entropy is defined as the mean value of $\ln_{q}\frac{1}{p_{i}}$. Moreover, under the usual linear constraints for the normalization and the energy mean value, it is optimized by a $\tilde{q}$-exponential factor, which precisely is the inverse function of the $\tilde{q}$-logarithmic function with the dual index $\tilde{q}=2-q$. One may ask how general is such a structure for trace-form entropic functionals. This is the question that was analyzed in [98].

The most general trace-form entropy $S_{G}$ can always be written as follows:

(49) $$S_{G}\left(\left\{p_{i}\right\}\right)=k\sum_{i=1}^{W}p_{i}\ln_{G}\frac{1}{p_{i}}\;,$$

where the generalized logarithm $\ln_{G}\left(z\right)$ (*z* being a real positive number) must be a monotonically increasing concave function for z>0, and also satisfy $\ln_{G}\left(1\right)=0$, among some other simple requirements [98]. The optimization of $S_{G}\left(\left\{p_{i}\right\}\right)$ under the usual linear constraints yields a distribution given by the generalized $\ln_{\tilde{G}}^{-1}\left(z\right)\equiv e_{\tilde{G}}\left(z\right)$, where $\tilde{G}$ denotes *dual* functions, duality being possibly defined in various manners. The simplest such manner is as follows:

(50) $$\ln_{\tilde{G}}\left(z\right)=-\ln_{G}\left(\frac{1}{z}\right)\;.$$

It is proved in [98] that the most general entropic functional (49) with duality given by (50) is precisely $S_{q}$.

### 2.12. Plastino-Plastino 2023 Connection with the Micro-Canonical Ensemble

Among trace form entropic measures, the $S_{q}$ non-additive entropies exhibit a special link with the micro-canonical ensemble [99,100]. Systems described by the micro-canonical ensemble usually have parts described by *q*-exponentials. That is, parts described by probability distributions optimizing the $S_{q}$ entropies. This happens when the number of micro-states of the rest of the system having energy less or equal to a given energy $E_{0}$ grows as a power of $E_{0}$. Here “the rest of the system” does not necessarily refer to a subsystem: it can refer, in the case of classical Hamiltonian systems, to a subset of the system’s canonical variables. For example, in classical non-relativistic scenarios, the kinetic energy of a system of *N* particles (interacting or not) is an homogeneous quadratic function of the momenta, implying that the volume in momentum-space grows in the above mentioned power-law fashion, the exponent depending on the number *N* of particles. Because of this power-law behavior, the marginal probability density for the configuration variables is a *q*-exponential of the total potential energy. The link between the micro-canonical ensemble and the $S_{q}$-canonical distributions is, in a sense, unique. Up to now, the $S_{q}$ entropy appears to be the only trace-form entropy exhibiting entropy-optimizing distributions that have been related to the micro-canonical treatment of concrete and physically relevant families of systems.

The unique character of this connection is particularly transparent in the classical non-relativistic regime: within that regime we can say that, to the extent that Nature prefers quadratic kinetic energies, it also prefers *q*-exponentials. In this regard, it is significative that *q*-exponentials are clearly discernible in a paper by Maxwell from 1879 [101,102] (see Equation (41) of [102]), which is one of the first papers ever discussing the micro-canonical ensemble, as we call it nowadays. Let us emphasize that Maxwell arrived to the *q*-exponentials without explicitly optimizing any entropic functional at all, just by assuming equal probabilities in the occupancy of the phase-space corresponding to a given total energy. To the best of our knowledge, probability distributions optimizing other of the non-logarithmic trace-form entropies discussed in the current research literature are not present in those pioneering works.

Before concluding this Subsection, let us mention that the above uniqueness might be not unrelated to the concept of *thermostat universal independence* introduced by Biro, Barnafoldi and Van in 2015 [103].

## 3. Closely Related Issues

### 3.1. The Values of the Entropic Indices Might Depend on the Class of States of the System

To illustrate the claim in the title of the present Subsection, let us focus on a specific example. The (1+1)-dimensional first-neighbor-interacting Ising ferromagnet in the presence of an external transverse field has, at its thermodynamical limit, a zero-temperature second-order critical point, i.e., a quantum critical point. At this point, the total entropy of this system vanishes since it is a *pure state*. Consider now a *L*-sized block (with L≫1) of this infinitely-sized system: it is in a *mixed state* and its nonvanishing BG entropy $S_{BG}\left(L\right)$ is given by [104,105]

(51) $$\frac{S_{BG}\left(L\right)}{k}∼\frac{c}{3}\ln L\;,$$

where c>0 is the *central charge* within the corresponding conformal field theory; for example, c=1/2 for the Ising ferromagnet, and c=1 for the XY ferromagnet. We are unaware of a rigorous proof determining the subdominant term, but strong indications [106] suggest the following behavior:

(52) $$\frac{S_{BG}\left(L\right)}{k}∼\frac{c}{3}\ln L+\ln b=\ln\left(bL^{c/3}\right)\;,$$

where b>1 is a constant. With the definition $W^{eff}\equiv bL^{c/3}$ (eff stands for *effective*), we have $\frac{S_{BG}\left(L\right)}{k}∼\ln W^{eff}\left(L\right)$. Consequently, *if the system was in an equal-probability state*, we could interpret $W^{eff}$ as the total number of possibilities. Then, we would have that, for L≫1,

(53) $$\frac{S_{q}\left(L\right)}{k}∼\ln_{q}W^{eff}\left(L\right)∼\ln_{q}\left(bL^{c/3}\right)=\frac{{\left[bL^{c/3}\right]}^{1-q}-1}{1-q}\propto L^{c(1-q)/3}\;\;\left(q<1\right)\;.$$

Thermodynamic extensivity of the entropy (generically required by the Legendre structure of thermodynamics [40]) would then imply $\frac{S_{q_{ent}}\left(L\right)}{k}\propto L$ with

(54) $$q_{ent}=1-\frac{3}{c}\;\;\;\left(c\geq 0\right)\;.$$

Let us emphasize that this result was obtained under the assumption of equal probabilities. It happens though that this assumption is *wrong* at the quantum critical point that we are focusing on! [106]. The correct result for this system is instead given by [105]

(55) $$q_{ent}=\frac{\sqrt{9+c^{2}}-3}{c}\;,$$

which definitively differs from $1-\frac{c}{3}$. Interestingly enough, however, the correct expression (55) asymptotically reproduces, in the c→∞ limit, the wrong expression (54), i.e., relation (55) implies $q_{ent}∼1-\frac{c}{3}$ (c→∞).

It is allowed to think that, perhaps quite generically, not only for $S_{q}$ but for other entropic functionals as well ($S_{q,\delta }$ [40] among others), the a priori assumption of equal probabilities for specific systems yields, at the relevant stationary state, the correct asymptotic behavior when approaching the BG limit (i.e., (q,δ)→(1,1) for $S_{q,\delta }$, for instance).

### 3.2. Entropic Functional vs. Entropy of a System

To be admissible, an entropic functional $S(\left\{p_{i}\right\})$ must be one and the same for all possible states, i.e., for all possible sets of the probabilities $\left\{p_{i}\right\}$ of a generic system. Such is, of course, the case of all entropic functionals that we have discussed up to now.

The Barrow proposal for entropy [107], noted $S_{\Delta }^{B}$ here, is *not* an entropic functional, but rather an expected value for black-holes or cosmological possibilities under the assumption of a rough external surface. Indeed, it is usually written as follows:

(56) $$S_{\Delta }^{B}∼A^{1+\Delta /2}\;,$$

or, equivalently, $S_{\Delta }^{B}∼L^{2+\Delta }$, where $A∼L^{2}$, *L* being the characteristic linear dimension of the system; for Δ=0, $S_{0}^{B}∼L^{2}$ recovers the usual Bekenstein-Hawking black-hole behavior; for Δ=1, $S_{1}^{B}∼L^{3}$ recovers the thermodynamically admissible entropy for a d=3 system; for 0<Δ<1 and also for Δ>1, the Barrow entropy $S_{\Delta }^{B}$ hopefully corresponds to a fractal-like black-hole surface. Summarizing, the Barrow entropy is extensive, i.e., thermodynamically admissible only for Δ=1. Indeed, for Δ<1 (Δ>1), $S_{\Delta }^{B}$ is subextensive (superextensive), thus violating the Legendre structure of thermodynamics.

On the other hand, let us focus on the entropic functional $S_{q,\delta }$ [40], defined as follows:

(57) $$S_{q,\delta }=k\sum_{i=1}^{W}p_{i}\;{\left[\ln_{q}\frac{1}{p_{i}}\right]}^{\delta }\;\;\left(q\in R,\;\delta \in R\right)\;,$$

which recovers, as particular instances, $S_{q,1}=S_{q}$ and $S_{1,\delta }=S_{\delta }\equiv k\sum_{i=1}^{W}p_{i}\;{\left[\ln\frac{1}{p_{i}}\right]}^{\delta }$. For equal probabilities, Equation (57) yields

(58) $$\frac{S_{1,\delta }}{k}={\left(\ln W\right)}^{\delta }$$

The Bekenstein-Hawking result $S_{BG}/k∼\ln W\propto A$ leads to

(59) $$\frac{S_{1,\delta }}{k}\propto A^{\delta }\;.$$

This expression can be identified with (56) through:

(60) $$\delta \equiv 1+\frac{\Delta }{2}\;.$$

This identity has produced in the literature some unfortunate confusion between the entropic functional $S_{\delta }$ [10] and the so-called Barrow entropy [107]. Let us emphasize, at this stage, that $S_{\Delta }^{B}$ is by no means analogous to the entropic functional $S_{\delta }\left(\left\{p_{i}\right\}\right)$ [40]. Indeed, the latter is an entropic functional applicable a priori to any system in any state, whereas, in contrast, the former has been specifically proposed for black holes at their thermal equilibrium state.

Recent observational data concerning dark energy physics have been interpreted [108] as being consistent with $S_{\delta }$ with δ=1.565. This value differs from δ=3/2 advanced in [40] under the three-fold hypothesis that (i) the system is a d=3 one with its surface being a d=2 one, (ii) entropic extensivity of $S_{\delta }$ for a *d*-dimensional system, and (iii) equal probabilities, which yields δ=d/(d−1). If the value of δ slightly different from 3/2 is taken as granted, then one or the other or even all three hypothesis could be inadequate. If we remind that, for a somewhat similar quantum system, the correct value of *q* differs from its value assuming equal probabilities (see Section 3.1), it cannot be excluded that δ=1.565 differing from δ=3/2 is rather caused by the failure of hypothesis (iii) and not necessarily by the failure of the other two hypothesis, which assume that the thermodynamically extensive entropy is based on the δ-entropic functional with the special value δ=d/(d−1).

An alternative explanation could of course be the failure of hypothesis (i), meaning that the system is a fractal-like one with say $\delta =\frac{d_{f}^{B}}{d_{f}^{S}}$, $d_{f}^{B}$ and $d_{f}^{S}$ being respectively the bulk and surface dimensionalities, not necessarily being given by $\left(d_{f}^{B},d_{f}^{S}\right)=\left(d,d-1\right)$. For example, if we impose $\frac{d_{f}}{d_{f}-1}=1.565$ with $\left(d_{f}^{B},d_{f}^{S}\right)=\left(d_{f},d_{f}-1\right)$ we obtain $d_{f}≃2.77$; if we instead impose say $3/d_{f}^{S}=1.565$ we obtain $d_{f}^{S}≃1.92$. Clearly, at this stage, the discrepancy 1.565 vs. 3/2 remains as an open, surely intriguing, question (Equation (57) yields, for equal probabilities, $\frac{S_{q,\delta }}{k}={\left(\ln_{q}W\right)}^{\delta }={\left[\frac{W^{1-q}-1}{1-q}\right]}^{\delta }∼\frac{W^{(1-q)\delta }}{{\left[1-q\right]}^{\delta }}\propto W^{(1-q)\delta }\;\;\;\left(W\to \infty \right)$. If this behavior is correct for a given system, then the value of the exponent (1−q)δ is to be preserved. Therefore, if a given *wrong* hypothesis (such as say equal probabilities) makes $\left(1-q_{wrong}\right)$ to be *larger* than $\left(1-q_{correct}\right)$ (as proved in Section 3.1), this implies, assuming a fixed value for $\left(1-q_{correct}\right)\delta_{correct}=\left(1-q_{wrong}\right)\delta_{wrong}$, that $\delta_{wrong}$ *smaller* than $\delta_{correct}$, which is precisely the inequality sense of 3/2 as compared to 1.565 !).

## 4. Summary

A plethora of entropic functionals (close to fifty) and their associated optimizing distributions are today available in the literature [16]. Among those, $S_{q}$ is by far the most frequently validated in natural, artificial and social complex systems up to now. Then, seeking for a deeper understanding, a natural question emerges: *in what senses is $S_{q}$ unique?* In the present review, we have listed (basically in chronological order) many such senses. In some cases, (Section 2.1, Section 2.3, Section 2.4, Section 2.6, Section 2.7, Section 2.8, Section 2.9, Section 2.11 and Section 2.12), the uniqueness of $S_{q}$ is established on rigorous grounds. In others (Section 2.2 and Section 2.5), it is conjectured on partial analytical arguments and/or strong numerical indications.

A related controversy has been briefly reviewed in Section 2.10.

Finally, in Section 3.1 and Section 3.2, we have illustrated that (i) the values of the entropic indices (for example, *q* for $S_{q}$ and δ for $S_{\delta }$) might depend on the class of states of the system (for example, assuming either equal or unequal probabilities, basically corresponding respectively to either a microcanonical or a canonical ensemble), and also that (ii) an entropic functional must be clearly distinguished from the same or from a different entropic functional *applied to a specific system in specific states*. Both are frequently referred to in the literature as “entropy”, but their mathematical role is sensibly distinct. Further senses along which $S_{q}$, or other entropic functionals, would be unique are certainly welcome.
